# Author Correction: An investigation of causal relationships between prediabetes and vascular complications

**DOI:** 10.1038/s41467-020-20663-6

**Published:** 2021-01-04

**Authors:** Pascal M. Mutie, Hugo Pomares-Millan, Naeimeh Atabaki-Pasdar, Nina Jordan, Rachel Adams, Nicole L. Daly, Juan Fernandes Tajes, Giuseppe N. Giordano, Paul W. Franks

**Affiliations:** 1 Genetic and Molecular Epidemiology Unit, Lund University Diabetes Centre, Department of Clinical Sciences, Clinical Research Centre, Lund University, Skåne University Hospital, Jan Waldenströms gata 35, Malmö, SE-20502 Sweden; 2 Regulatory Affairs Intelligence, Novo Nordisk A/S, Copenhagen, Denmark; 3 Regulatory Affairs—Neuroscience and Cardiovascular Metabolism, Janssen, High Wycombe, UK; 4 Department of Public Health and Clinical Medicine, Section for Medicine, Umeå University, Umeå, Sweden; 5 Department of Nutrition, Harvard T.H. Chan School of Public Health, Boston, MA USA

**Keywords:** Cardiovascular diseases, Epidemiology, Genetics research

Correction to: *Nature Communications* 10.1038/s41467-020-18386-9, published online 14 September 2020.

The original version of this Article contained an error in the second sentence of the introduction, in which the measurement units of HbA1c incorrectly read ‘mmol L^−1^’ rather than the correct ‘mmol mol^−1^’. This has been corrected in both the PDF and HTML versions of the Article.

