# Supplementary material for: An investigation of causal relationships between prediabetes and vascular complications
Source: Nat Commun. 2020 Sep 14;11:4592. doi: 10.1038/s41467-020-18386-9 (PMC7490420; doi:10.1038/s41467-020-18386-9)
Supplement: Supplementary file 1 — Supplementary Information [file 41467_2020_18386_MOESM1_ESM.docx]

SUPPLEMENTARY INFORMATION

AN INVESTIGATION OF CAUSAL RELATIONSHIPS BETWEEN PREDIABETES AND VASCULAR COMPLICATIONS

Mutie et al (2020)

SUPPLEMENTARY NOTE 1

We performed sensitivity analyses to investigate the influence of a single study on the pooled estimates. We recalculated the pooled estimates removing each study at a time in each run. From these analyses, the results of the meta-analysis do not seem to be driven by a single study for coronary artery disease (CAD), stroke or chronic kidney disease (CKD). Moreover, the magnitude and direction of effect estimates remain materially unchanged when all studies are included.

SUPPLEMENTARY FIGURES

Supplementary Figure 1. Meta-analysis for CAD with one study omitted at a time. Data are represented as relative risks (RR) and 95% confidence interval (95% CI). The central points represent the effect estimates (RR) while the horizontal bars represent the respective 95% CI. Combined RR = 1.16, 95% CI 1.08, 1.25 (n = 21 studies)

Supplementary Figure 2. Meta-analysis for Stroke with one study omitted at a time. Data are represented as relative risks (RR) and 95% confidence interval (95% CI). The central points represent the effect estimates (RR) while the horizontal bars represent the respective 95% CI. Combined RR = 1.11, 95% CI 1.01, 1.21, n = 14 studies

Supplementary Figure 3. Meta-analysis for CKD with one study omitted at a time. Data are represented as relative risks (RR) and 95% confidence interval (95% CI). The central points represent the effect estimates (RR) while the horizontal bars represent the respective 95% CI. Combined RR = 1.06, 95% CI 0.99, 1.12, n = 8 studies.

SUPPLEMENTARY NOTE 2

We performed sensitivity analyses using the two-sample Mendelian randomization (MR) method to strengthen our findings by investigating the relationship between CAD (which was significantly associated with prediabetes in our main analyses) and cholesterol / lipid levels as well as with two-hour glucose levels. GWAS summary data for lipids/cholesterol were acquired from the Global lipids genetics consortium^1^ and BMI data were extracted from the Giant consortium portal.^2^ Two-hour glucose GWAS summary statistics data were acquired from the MAGIC consortium online portal^3^. The quality control (QC) procedures outlined in the manuscript were followed to get final data sets for two-sample MR analysis.

*Association between 2-hr glucose and risk of CAD*

After the routine quality control filtering, only two SNPs that are genome-wide significantly associated with 2hr glucose and not associated with T2D at nominal significance remained. This number was below that required to perform meaningful two-sample MR statistical tests and so no further analyses were attempted.

We also assessed the causal association between 2-hr glucose and risk of CAD using all 2-hr glucose SNPs that were genome-wide significant, irrespective of whether they were or were not associated with T2D. After QC, the resultant number was seven uncorrelated SNPs. There was no significant causal relationship detected between 2-hr glucose and risk of CAD however, there was evidence of horizontal pleiotropy, P_EggerIntercept_ = 0.00 (Robust IVW), see supplementary table 1.

*Association between prediabetes only and other CAD risk factors*

We tested the causal association with several additional risk factors for CAD (LDL cholesterol, HDL cholesterol, Total Cholesterol, Triglycerides and BMI). Genetically determined fasting glucose was not causally related to any of these traits, see supplementary table 2.

# SUPPLEMENTARY TABLES

| Supplementary Table 1. Causal association between 2-hr glucose and the risk of CAD, n = 7 SNPs | | | | |
| --- | --- | --- | --- | --- |
| Method | OR | Lower 95% CI | Upper 95% CI | P-value |
| Weighted median | 0.99 | 0.86 | 1.13 | 0.83 |
| IVW | 1.00 | 0.87 | 1.17 | 0.96 |
| Robust IVW | 1.00 | 0.85 | 1.16 | 0.96 |
| MR-Egger | 0.60 | 0.28 | 1.30 | 0.20 |
| Intercept _MR-Egger_ | 1.05 | 0.98 | 1.12 | 0.19 |
| Robust MR-Egger | 0.60 | 0.40 | 0.88 | 0.01 |
| Intercept _Robust MR-Egger_ | 1.05 | 1.01 | 1.08 | 0.00 |

IVW = Inverse-variance weighted, OR = Odds Ratio, CI = Confidence Interval.

Results are from two-sample Mendelian Randomization analysis and P-values are two-sided.

Results are unadjusted for multiple comparisons.

| Supplementary Table 2. Causal association between prediabetes only and LDL cholesterol (n = 28 SNPs) | | | | |
| --- | --- | --- | --- | --- |
| Method | Estimate | Lower 95% CI | Upper 95% CI | P-value |
| Weighted median | 1.07 | 0.98 | 1.17 | 0.13 |
| IVW | 1.08 | 0.95 | 1.24 | 0.25 |
| Robust IVW | 1.04 | 0.99 | 1.1 | 0.09 |
| MR-Egger | 1.09 | 0.85 | 1.39 | 0.52 |
| Intercept _MR-Egger_ | 1.00 | 0.99 | 1.01 | 0.97 |
| Robust MR-Egger | 1.08 | 1.00 | 1.17 | 0.05 |
| Intercept _Robust MR-Egger_ | 1.00 | 1.00 | 1.00 | 0.44 |

IVW = Inverse-variance weighted, Estimate = Beta (unit change in biomarker level).

CI = Confidence Interval. Results are from two-sample Mendelian Randomization

analysis and P-values are two-sided. Results are unadjusted for multiple comparisons.

| Supplementary Table 3. Causal association between prediabetes only and HDL levels (n = 28 SNPs) | | | | |
| --- | --- | --- | --- | --- |
| Method | Estimate | Lower 95% CI | Upper 95% CI | P-value |
| Weighted median | 1.01 | 0.92 | 1.11 | 0.82 |
| IVW | 1.08 | 0.92 | 1.27 | 0.33 |
| Robust IVW | 0.98 | 0.89 | 1.08 | 0.74 |
| MR-Egger | 0.87 | 0.65 | 1.14 | 0.31 |
| Intercept _MR-Egger_ | 1.01 | 1.00 | 1.02 | 0.06 |
| Robust MR-Egger | 0.97 | 0.76 | 1.24 | 0.84 |
| Intercept _Robust MR-Egger_ | 1.00 | 0.99 | 1.01 | 0.92 |

IVW = Inverse-variance weighted, Estimate = Beta (unit change in biomarker level). Results are from two-sample Mendelian Randomization analysis and P-values are two-sided. Results are unadjusted for multiple comparisons.

| Supplementary Table 4. Causal association between prediabetes only and triglycerides (n = 28 SNPs) | | | | |
| --- | --- | --- | --- | --- |
| Method | Estimate | Lower 95% CI | Upper 95% CI | P-value |
| Weighted median | 0.99 | 0.91 | 1.07 | 0.77 |
| IVW | 0.88 | 0.74 | 1.05 | 0.17 |
| Robust IVW | 0.96 | 0.88 | 1.04 | 0.29 |
| MR-Egger | 1.13 | 0.83 | 1.52 | 0.44 |
| Intercept _MR-Egger_ | 0.99 | 0.98 | 1.00 | 0.06 |
| Robust MR-Egger | 1.09 | 0.94 | 1.25 | 0.26 |
| Intercept _Robust MR-Egger_ | 1.00 | 0.99 | 1.00 | 0.18 |

IVW = Inverse-variance weighted, Estimate = Beta (unit change in biomarker level).

CI = Confidence Interval. Results are from two-sample Mendelian Randomization

analysis and P-values are two-sided. Results are unadjusted for multiple comparisons.

| Supplementary Table 5. Causal association between prediabetes only and total cholesterol (n = 28 SNPs) | | | | |
| --- | --- | --- | --- | --- |
| Method | Estimate | Lower 95% CI | Upper 95% CI | P-value |
| Weighted median | 1.04 | 0.95 | 1.14 | 0.45 |
| IVW | 1.07 | 0.94 | 1.23 | 0.31 |
| Robust IVW | 1.04 | 0.98 | 1.11 | 0.22 |
| MR-Egger | 1.08 | 0.84 | 1.38 | 0.55 |
| Intercept _MR-Egger_ | 1.00 | 0.99 | 1.01 | 0.96 |
| Robust MR-Egger | 1.09 | 0.98 | 1.20 | 0.10 |
| Intercept _Robust MR-Egger_ | 1.00 | 0.99 | 1.00 | 0.44 |

IVW = Inverse-variance weighted, Estimate = Beta (unit change in biomarker level).

CI = Confidence Interval. Results are from two-sample Mendelian Randomization

analysis and P-values are two-sided. Results are unadjusted for multiple comparisons.

| Supplementary Table 6. Causal association between prediabetes only and BMI. n = 28 SNPs | | | | |
| --- | --- | --- | --- | --- |
| Method | Estimate | Lower 95% CI | Upper 95% CI | P-value |
| Weighted median | 0.98 | 0.94 | 1.02 | 0.37 |
| IVW | 0.98 | 0.92 | 1.05 | 0.62 |
| Robust IVW | 0.98 | 0.96 | 1.00 | 0.08 |
| MR-Egger | 0.92 | 0.81 | 1.04 | 0.19 |
| Intercept _MR-Egger_ | 1.00 | 1.00 | 1.01 | 0.22 |
| Robust MR-Egger | 0.95 | 0.87 | 1.02 | 0.17 |
| Intercept _Robust MR-Egger_ | 1.00 | 1.00 | 1.00 | 0.44 |

IVW = Inverse-variance weighted, Estimate = Beta (unit change in biomarker level).

CI = Confidence Interval. BMI = Body Mass Index. Results are from two-sample

Mendelian Randomization analysis and P-values are two-sided.

Results are unadjusted for multiple comparisons.

SUPPLEMENTARY REFERENCES:

1 Willer, C. J. *et al.* Discovery and refinement of loci associated with lipid levels. *Nat Genet* **45**, 1274-1283, doi:10.1038/ng.2797 (2013).

2 Yengo, L. *et al.* Meta-analysis of genome-wide association studies for height and body mass index in approximately 700000 individuals of European ancestry. *Hum Mol Genet* **27**, 3641-3649, doi:10.1093/hmg/ddy271 (2018).

3 Morris, A. P. *et al.* Large-scale association analysis provides insights into the genetic architecture and pathophysiology of type 2 diabetes. *Nature genetics* **44**, 981-990, doi:10.1038/ng.2383 (2012).
