# Supplementary material for: An investigation of causal relationships between prediabetes and vascular complications
Source: Nat Commun. 2020 Sep 14;11:4592. doi: 10.1038/s41467-020-18386-9 (PMC7490420; doi:10.1038/s41467-020-18386-9)
Supplement: Supplementary file 3 — Description of Additional Supplementary files [file 41467_2020_18386_MOESM3_ESM.docx]

DESCRIPTION OF ADDITIONAL SUPPLEMENTARY FILES

File name: Supplementary Data 1

Description: This file contains the search string used to retrieve observational studies for meta-analysis.

File name: Supplementary Data 2

Description: This file contains the following data

1. All studies used in the meta-analysis, with the columns:
   - 1. Name of first author, Year
     2. Prediabetes definition
     3. Prevalence %
     4. N (sample size)
     5. Women %
     6. Age mean (mean age of participants)
     7. SD or range
     8. Follow-up mean (yr)
     9. Outcome
     10. RR
     11. 95% LCI
     12. 95% UCI
     13. Outcome definition
     14. Outcome ascertainment
     15. Adjusted variables
     16. Adequacy of adjustment
     17. Baseline outcome excluded (Yes/No)
     18. Possibility of enrolling patients with diabetes (Yes/No)
     19. Possibility of developing diabetes during study (Yes/No)
     20. Developed diabetes at follow-up (n)
     21. HR: Incidence density (events/ person-time)
2. The studies’ quality assessment scores in the Newcastle – Ottawa Scale
3. Subgroup analysis of observational studies
