# Supplementary material for: An investigation of causal relationships between prediabetes and vascular complications
Source: Nat Commun. 2020 Sep 14;11:4592. doi: 10.1038/s41467-020-18386-9 (PMC7490420; doi:10.1038/s41467-020-18386-9)
Supplement: Supplementary file 6 — Reporting Summary [file 41467_2020_18386_MOESM6_ESM.pdf]

nature research

Corresponding author(s): Paul W. Franks

Last updated by author(s): Jun 25, 2020

Reporting Summary

Nature Research wishes to improve the reproducibility of the work that we publish. This form provides structure for consistency and transparency in reporting. For further information on Nature Research policies, see our Editorial Policies and the Editorial Policy Checklist.

Statistics

For all statistical analyses, confirm that the following items are present in the figure legend, table legend, main text, or Methods section.

n/a	Confirmed	
☐	☒	The exact sample size (*n*) for each experimental group/condition, given as a discrete number and unit of measurement
☐	☒	A statement on whether measurements were taken from distinct samples or whether the same sample was measured repeatedly
☐	☒	The statistical test(s) used AND whether they are one- or two-sided<br>*Only common tests should be described solely by name; describe more complex techniques in the Methods section.*
☐	☒	A description of all covariates tested
☐	☒	A description of any assumptions or corrections, such as tests of normality and adjustment for multiple comparisons
☐	☒	A full description of the statistical parameters including central tendency (e.g. means) or other basic estimates (e.g. regression coefficient) AND variation (e.g. standard deviation) or associated estimates of uncertainty (e.g. confidence intervals)
☐	☒	For null hypothesis testing, the test statistic (e.g. *F*, *t*, *r*) with confidence intervals, effect sizes, degrees of freedom and *P* value noted<br>*Give P values as exact values whenever suitable.*
☒	☐	For Bayesian analysis, information on the choice of priors and Markov chain Monte Carlo settings
☐	☒	For hierarchical and complex designs, identification of the appropriate level for tests and full reporting of outcomes
☐	☒	Estimates of effect sizes (e.g. Cohen's *d*, Pearson's *r*), indicating how they were calculated

Our web collection on statistics for biologists contains articles on many of the points above.

Software and code

Policy information about availability of computer code

Data collection	No software or special code was used for data collection.
Data analysis	Data analyses were performed using STATA software v.13 (commercially available, Stata Corp LP, College Station, TX) and R software v. 3.6.1 (publicly available, https://cran.r-project.org/). Specific R packages used for Mendelian Randomization are TwoSampleMR, MendelianRandomization and MRPRESSO.

For manuscripts utilizing custom algorithms or software that are central to the research but not yet described in published literature, software must be made available to editors and reviewers. We strongly encourage code deposition in a community repository (e.g. GitHub). See the Nature Research guidelines for submitting code & software for further information.

Data

Policy information about availability of data

All manuscripts must include a data availability statement. This statement should provide the following information, where applicable:

- Accession codes, unique identifiers, or web links for publicly available datasets
- A list of figures that have associated raw data
- A description of any restrictions on data availability

Data used in meta-analysis of observational studies were extracted from the PUBMED database using a search string detailed in Supplementary_Data_1.
Data (GWAS summary statistics) used in Mendelian Randomization analyses were retrieved from the following publicly available data repositories:
Coronary Artery Disease (CAD):
URL: https://data.mendeley.com/datasets/gbbsrpx6bs/1#file-67c31537-5906-40bb-9820-8764b1554666 (DOI: 10.17632/gbbsrpx6bs.1)
Dataset name: CAD_META.gz

Chronic Kidney Disease (CKD):
URL: http://ckdgen.imbi.uni-freiburg.de/
Dataset: CKD overall European ancestry (filed under Wuttke et al. 2019 publication)

Type 2 diabetes (T2D):
URL: https://www.diagram-consortium.org/downloads.html
Dataset: T2D GWAS meta-analysis - Unadjusted for BMI, filed under Mahajan et al. (2018b)

Fasting Glucose, 2-hr glucose and HbA1c:
URL: https://www.magicinvestigators.org/downloads/
Fasting and 2-hr glucose datasets: Filed under Metabochip replication datasets, zipped file containing both datasets = ftp://ftp.sanger.ac.uk/pub/magic/MAGIC_Metabochip_Public_data_release_25Jan.zip.
HbA1c dataset: ftp://ftp.sanger.ac.uk/pub/magic/HbA1c_METAL_European.txt.gz

Stroke:
URL: https://megastroke.org/download.html
Dataset: MEGASTROKE_data.zip (accessible after agreeing to terms of use and submitting a brief project description. The zipped folder contains separated datasets for Any Stroke, Any Ischemic Stroke, Large Artery Stroke, Cardioembolic Stroke and Small Vessel Stroke)

Lipids
URL: http://csg.sph.umich.edu/willer/public/lipids2013/
Datasets: Filed under ”RESULT FILES”, subheading “JOINT ANALYSIS OF METABOCHIP AND GWAS DATA” names of files = LDL Cholesterol, HDL Cholesterol, Triglycerides and Total Cholesterol.

Body Mass Index
URL: http://portals.broadinstitute.org/collaboration/giant/index.php/GIANT_consortium_data_files
Dataset: Filed under “BMI and Height GIANT and UK BioBank Meta-analysis Summary Statistics”, name of file = Meta-analysis Wood et al + UKBiobank 2018 GZIP

Raw data used to generate all figures and tables in the current study have been provided in Source_Data file.

There are no restrictions, as at the time of publication, to any of the data used in this study. All data are publicly available and accessible via the links provided above. In addition, we have provided all the data generated and used in the current study in the Source_Data file.

Field-specific reporting

Please select the one below that is the best fit for your research. If you are not sure, read the appropriate sections before making your selection.

- [x] Life sciences
- [ ] Behavioural & social sciences
- [ ] Ecological, evolutionary & environmental sciences

For a reference copy of the document with all sections, see nature.com/documents/nr-reporting-summary-flat.pdf

Life sciences study design

All studies must disclose on these points even when the disclosure is negative.

Sample size

While we did not perform formal sample size calculations, the resulting sample sizes in both the observational meta-analysis and MR analyses were adequate for analysis. In both cases, the same underlying principle applies, i.e. pooling individual study (or genetic variant) effect sizes weighted by a relevant parameter, in this case the inverse of the variance. According to Valentine et al (2010, DOI: 10.3102/1076998609346961), one technically needs two studies for meta-analysis, but the results may be highly biased. Statistically, one can derive a statistical tests on a sample of more than 3 but bias and low power will affect the results. Therefore, we deem our sample sizes to be adequate for our analyses.

Data exclusions

In the observational meta-analysis, studies extracted using the search string were excluded if; they were not proper research articles (e.g. letters, protocols, e.t.c.), the title and abstract were deemed not relevant to the current study, they did not report outcomes relevant to this study, they did not report relevant measures of association, the study design was not appropriate, there was no exposure assessment at baseline and the study population had outcome at baseline. In Mendelian Randomization analyses, genetic variants were excluded if they had a high P-value (P > 5e-8), were in high linkage disequillibrium (r^2 > 0.2) or if they were pallindromic and unresolved.

Replication

Experimental replication was not relevant in our current study. The study design utilized observational data for meta-analysis and GWAS summary data for causal inference analyses and therefore does not meet the requirements for replication.

Randomization

Randomization was not relevant in our current study. The study design utilized observational data for meta-analysis and GWAS summary data for causal inference analyses and therefore does not meet the requirements for randomization.

Blinding

Blinding was not relevant in our current study. The study design utilized observational data for meta-analysis and GWAS summary data for causal inference analyses and therefore does not meet the requirements for blinding.

Reporting for specific materials, systems and methods

We require information from authors about some types of materials, experimental systems and methods used in many studies. Here, indicate whether each material, system or method listed is relevant to your study. If you are not sure if a list item applies to your research, read the appropriate section before selecting a response.

Materials & experimental systems

n/a	Involved in the study
☒	☐ Antibodies
☒	☐ Eukaryotic cell lines
☒	☐ Palaeontology and archaeology
☒	☐ Animals and other organisms
☒	☐ Human research participants
☒	☐ Clinical data
☒	☐ Dual use research of concern

Methods

n/a	Involved in the study
☒	☐ ChIP-seq
☒	☐ Flow cytometry
☒	☐ MRI-based neuroimaging
